# Assessment of olfactory and gustatory functions in COVID-19 patients

**DOI:** 10.3906/sag-2102-290

**Published:** 2021-10-21

**Authors:** Muammer Melih ŞAHİN, Eray UZUNOĞLU, Mücahit YALÇIN, Gökçen CESUR, Mehmet YILDIZ, Pınar AYSERT YILDIZ, Hasan Selçuk ÖZGER, Süleyman CEBECİ, Recep KARAMERT, Mehmet DÜZLÜ, Hakan TUTAR, Murat DİZBAY, Alper CEYLAN

**Affiliations:** 1 Department of Otorhinolaryngology/Head and Neck Surgery, Faculty of Medicine, Gazi University, Ankara Turkey; 2 Department of Infectious Disease and Clinical Microbiology, Faculty of Medicine, Gazi University, Ankara Turkey

**Keywords:** COVID-19, anosmia, ageusia, sniffin sticks

## Abstract

**Background/aim:**

This study aims to evaluate of olfactory and gustatory functions of COVID-19 patients and possible risk factors for olfactory and gustatory dysfunctions.

**Materials and methods:**

The cross-sectional study included adult patients who were diagnosed with COVID-19 in Gazi University Hospital between April 2020 and June 2020. Volunteered patients participated in a survey in which olfactory and gustatory functions and various clinical information were questioned. Sinonasal Outcome Test-22 was also administrated to all patients.

**Results:**

A hundred and seventy-one patients participated in this study. Olfactory and gustatory dysfunctions rates were 10.5% (n: 18) and 10.5% (n: 18), respectively. Patients without any symptom other than smell and taste dysfunctions were clustered as group 1 and patients who are clinically symptomatic were clustered as group 2. Olfactory dysfunction occurred in 8% of group 1 and 17.4% of group 2 (p = 0.072). Gustatory dysfunction rate of smokers was 19.7% and significantly higher than gustatory dysfunction rate of nonsmokers (5.5%) (p = 0.007). Twenty-seven-point-eight percent of the patients with olfactory dysfunction (n = 5) were male and 72.2% (n: 13) were female. Sex did not show significant effect on rate of olfactory dysfunction. Twenty-five patients participated in psychophysical olfactory function test. No participant reported olfactory dysfunction at the time of test. Of the participants, 64% (n: 16) were normosmic and 36% (n: 9) were hyposmic according to Sniffin’ Stick test.

**Conclusion:**

Olfactory and gustatory dysfunctions are more common in patients who are clinically symptomatic than those diagnosed during contact tracing. Objective tests may show that frequency of olfactory dysfunction is greater than frequency of self-reported olfactory dysfunction.

## 1. Introduction

Corona virus disease 2019 (COVID-19) is a pandemic that emerged from East Asia in late 2019 and rapidly spread to the rest of the world and caused by severe acute respiratory syndrome Coronavirus 2 (SARS-CoV-2) [1]. The disease is held responsible for more than 2 million deaths worldwide and still poses a threat to the public health in most of the countries. The most common manifestations of the disease are fever, coughing, sore throat, and dyspnea [1,2]. 

Smell and taste dysfunctions have been frequently reported since the onset of the disease. In one of the very first reports of the neurologic manifestation of the disease, Mao et al. reported anosmia and ageusia in 5.1% and 5.6% of the cases, respectively [3]. Although incidences of taste and smell dysfunctions vary among the reports, Lechien et al. reported that 85.6% of the patients in Europe suffered from olfactory dysfunction and 88.8% had gustatory dysfunction [4]. Early reports claimed chemosensory disorders may be the initial signs of the disease, especially in the asymptomatic patients [4–7]. Sudden onset of anosmia and ageusia within 24–48 h is reported to be highly predictive for the disease and these symptoms often occur within 5 days from the onset of disease [8].

Alternation of chemosensory functions due to a viral infection is not a new phenomenon for otolaryngologists. In adults, 40% of anosmia cases are caused by a viral upper respiratory tract infection [9]. Common pathogens of upper respiratory tract, rhinovirus, parainfluenza, Epstein-Barr virus, and coronavirus are known to cause olfactory and gustatory dysfunctions [10,11]. On the other hand, olfactory dysfunction pathogenesis in COVID-19 seems to be on a different aspect since anosmia may occur without rhinorrhea or any other findings related to upper respiratory tract infection [4,8]. Lechien et al. reported the occurrence of anosmia or hyposmia in the absence of rhinorrhea and nasal congestion in 79.7% of the patients [4]. To explain mechanism of chemosensory disorders in COVID-19 several hypotheses were raised. SARS-Cov-2 binds directly the angiotensin converting enzyme 2 (ACE 2) cell receptors. ACE2 receptors take place frequently on olfactory epithelium and on oral cavity mucosa particularly on the tongue but may also be detected on glial cells and neurons of central nervous system as well [12–14]**.** Epithelial damage of nasal and oral mucosa may have a role in olfactory and gustatory dysfunctions [15,16]. SARS-CoV-2 is shown to be neuro-invasive and invasion of the olfactory nerve and trigeminal nerve may cause olfactory and gustatory dysfunctions [17]. Another hypothesis claims that central nervous system involvement with focal encephalitis in olfactory and gustatory cortex may be the cause of olfactory and gustatory dysfunctions. The detection of viral RNA in cerebrospinal fluid of the patients may support this hypothesis [3].

Even though the mechanism of olfactory and gustatory dysfunctions in COVID-19 patients is not fully discovered, these symptoms keep importance for suspecting of disease and early diagnosis. In this study, we aim to evaluate olfactory and gustatory dysfunctions of COVID-19 patients with a subjective self-reported questionnaire. We also investigated the long-term effects of COVID-19 on olfaction with an objective psychophysical test. 

## 2. Material and methods 

### 2.1. Study design

This cross-sectional study was approved by Gazi University Ethical committee of clinical research. The study included adult patients who diagnosed with COVID-19 in Gazi University Hospital between April 2020 and June 2020. The diagnosis was made with a positive SARS-Cov-2 PCR test. The patients were invited to this study by phone. All participants provided informed consent. In the survey, each patient was interviewed about basic demographic info, the time of diagnosis, hospitalization time, presence of olfactory and gustatory dysfunctions, onset of these symptoms (days before or days after diagnosis), recovery of olfactory and gustatory dysfunctions, history of any rhinologic surgery. Sinonasal Outcome Test-22 (SNOT-22) was also administrated to all patients. Patients with the history of smell or taste dysfunctions prior to COVID-19 and history of previous rhinologic surgery were excluded from the study. Two hundred and ninety-eight patients had been diagnosed with COVID-19 between April 2020 and June 2020. Ninety-six patients refused to participate in the study. Five patients with olfactory dysfunction prior to COVID-19 and 26 patients with a history of rhinologic surgery were excluded from the study. A hundred and seventy-one patients met the inclusion criteria.

Information on disease severity and treatment modality was obtained from the patients’ files. The patients were divided into groups according to disease severity. Patients without any symptom other than smell and taste dysfunction were clustered as group 1 and patients who are clinically symptomatic were clustered as group 2. Group 2 was further divided in two groups as the patients with pneumonia were clustered as group 2b and the others were clustered as group 2a.

### 2.2. Psychophysical olfactory evaluation

Patients whose recovery of the disease was shown with two consecutive negative SARS-CoV2 tests were invited to the clinic for objective assessment of olfaction with Sniffin’ stick test battery (Sniffin’ Sticks, Burghart GmbH, Wedel, Germany). Twenty-five patients volunteered to participate. The test was performed within 45 days and at least 30 days after the diagnosis of COVID-19. No participant had a history of head trauma or another episode of upper respiratory tract infection after the diagnosis of COVID-19. The tests were performed as previously described by Rumeau et al. [18]. All the tests were performed in a well-ventilated, odor-free room. The investigator who performed the test used personal protective equipment during the procedure. An interval of at least 8 h took place between tests to prevent spread of the disease. Threshold (T), Discrimination (D), Identification (I), and global scores (TDI) were recorded for each patient. Patients with a TDI score lower than 15 are considered anosmic, patients with a score between 15 and 30 are considered as hyposmic, and patients with a score equal to or higher than 30 are considered normosmic. 

### 2.3. Statistical analysis

Statistical analysis was performed with IBM SPSS v 22.0 (IBM Corp, Armonk, New York). The Shapiro–Wilk test was used for assessing normality. To display demographic information, mean ± standard deviation (SD) was used for normally distributed variables and median (min–max) for nonnormally distributed variables. Chi-square was used for categorical data. The Student t-test and the Mann–Whitney U test were used to compare the normal and nonnormally distributed data between two groups, respectively. Spearman’s rank correlation coefficient was used for correlation of nonnormally distributed data. The level of statistical significance was set at p ≤ 0.05 with a 95% confidence interval.

## 3. Results

### 3.1. Demographic findings and the survey

A hundred and seventy-one patients participated in this study. Of the participants, 58.5% (n: 100) were female and 41.5% (n: 71) were male. The median age of the participants was 36 (min–max: 18–71). Of the patients, 35.7% (n: 61) were smokers and 12.9% (n: 22) were previously diagnosed with allergic rhinitis. Participants’ demographic and clinic information is summarized on Table 1. 

**Table 1:** Demographic and clinical information of patient participated in survey.

	n	%
Sex		
Male	71	41.5
Female	100	58.5
Disease Groups		
Group 1	125	73.1
Group 2a	35	20.5
Group 2b	11	6.4
Allergic rhinitis	22	12.9
Smoking	61	35.7
Olfactory dysfunction	18	10.5
Gustatory dysfunction	18	10.5

The patients were clustered in three groups according to disease severity, 73.1% of the patients (n: 125) were in group 1 (asymptomatic other than smell or taste dysfunction), 20.5% (n: 35) were in group 2a (symptomatic disease without pneumonia), and 6.4% (n: 11) were in group 2b (with pneumonia). In total, 26.9% of the patients (n: 46) were clinically symptomatic (group 2). 

Patients who suffered from olfactory dysfunction and gustatory dysfunction comprised 10.5% (n: 18) and 10.5 (n: 18) of all the patients, respectively. Olfactory and gustatory dysfunctions were present together in 6.4% of the patients (n: 11). Clinical information of patients with and without olfactory dysfunction is summarized in Table 2, and clinical information of patients with and without gustatory dysfunction is summarized in Table 3. Olfactory dysfunction occurred before diagnosis in 8 patients. The median time between onset of olfactory dysfunction and diagnosis was 2.5 days (min–max: 1–5). In 10 of the patients, olfactory dysfunction occurred after diagnosis with a median interval of 1 day (min–max: 1–7). All but one patient recovered from olfactory dysfunction in a median time of 7 days (min–max: 0–30). One patient was still suffering from olfactory dysfunction 45 days after diagnosis. Gustatory dysfunction occurred before diagnosis in 8 participants within a median time of 2 days (min–max: 1–5) and after diagnosis in 10 participants within a median time of 1 day (min–max: 1–3). Gustatory dysfunction resolved in 16 of the patients within a median time of 8.5 days (min–max: 1–30). 

**Table 2:** Comparison of patients with and without olfactory dysfunction.

	With olfactorydysfunction (18)	Without olfactorydysfunction (153)	p
Age median (min–max)	39.5 (18–55)	36 (19–71)	0.772
Sex			
Male	27.8% (5)	43.1% (66)	0.159
Female	72.2% (13)	56.9% (87)	
Allergic rhinitis	27.8% (5)	11.1% (17)	0.061
Smoking habit	44.4% (8)	34.6% (53)	0.411

Olfactory dysfunction occurred in 8% of group 1 and 17.4% of group 2**.** Olfactory dysfunction rate in group 1 was lower than that in group 2 but that was not statistically significant (p = 0.072). Olfactory dysfunction rates in groups 2a and 2b were 17.1% and 18.2%, respectively. Gustatory dysfunction occurred in 8% of group 1 and in 17.4% of group 2 and that was not statistically significant (p = 0.072). Gustatory dysfunction rates in group 2a and 2b were 17.1% and 18.2%, respectively. 

The median ages of the patients who suffered from olfactory dysfunction and who did not were 39.5 years (min–max: 18–55) and 36 years (min–max: 19–71), respectively (p > 0.005).

Patients with a previous diagnosis of allergic rhinitis had a higher olfactory dysfunction rate compared to the other patients (22.7% and 8.7%, respectively) but that was not statistically significant (p > 0.05). Gustatory dysfunction rates were also higher in the patients with allergic rhinitis (22.7% vs 8.7%) (p > 0.05). 

Olfactory dysfunction rates in smokers and nonsmokers were 13.1% and 9.1%, respectively. Gustatory dysfunction rate of smokers was 19.7% and significantly higher than gustatory dysfunction rate of nonsmokers (5.5%) (p = 0.007).

Olfactory dysfunction occurred in 7.0% of the male patients and 13.0% of the female patients but the difference was not significant (p = 0.16).

Mean recovery time of olfactory dysfunction was 17.3 ± 12.3 days in group 2 and 9.2 ± 8.2 days in group 1. The difference was insignificant (p > 0.05). The mean recovery time of olfactory dysfunction of smokers was 12.6 ± 11.5 days and was similar with the mean recovery time of nonsmokers (12.4 ± 10.3 days). 

### 3.2. SNOT-22 questionnaire

The mean SNOT-22 score of all the patients was 11.6 ± 13.2 (min–max: 0–59). In groups 1, 2a, and 2b, the mean scores were 10.3 ± 11.7, 17.9 ± 17.2, 6.73 ± 9.9, respectively. There was not statistically significant difference between SNOT-22 scores of groups 1 and 2 (p = 0.19). 

The mean SNOT-22 score of patients who suffered from olfactory dysfunction was 20 ± 13.4 and the mean score of patients who did not was 10.6 ± 12.9. The difference was statistically significant. (p = 0.001) (Table 4). The mean SNOT 22 scores of patients who had gustatory dysfunction and who did not were 27.2 ± 14.9 and 9.8 ± 11.8, respectively. The difference was also statistically significant p < 0.001). Analysis of the correlation between SNOT-22 scores and recovery times (days) of olfactory and gustatory dysfunctions did not show significance (p > 0.05 R: 0.315). 

**Table 4:** SNOT-22 questionnaire and mean scores for each question.

Question	All	With olfactory Dysfunction	Without olfactory Dysfunction	p
1. Need to blow nose	0.36 ± 0.9	0.83 ± 1.2	0.31 ± 0.8	0.02*
2. Nasal Obstruction	0.56 ± 1	1.39 ± 1.3	0.46 ± 0.9	0.001*
3. Sneezing	0.63 ± 1	1.28 ± 1.4	0.55 ± 1	0.007*
4. Runny nose	0.46 ± 0.9	0.61 ± 1.2	0.44 ± 0.9	>0.05
5. Cough	0.61 ± 1	0.61 ± 1	0.61 ± 1	>0.05
6. Postnasal discharge	0.50 ± 1	0.83 ± 1.3	0.46 ± 0.9	>0.05
7. Thick nasal discharge	0.15 ± 0.5	0.50 ± 0.9	0.10 ± 0.4	0.005*
8. Ear fullness	0.19 ± 0.6	0.44 ± 0.9	0.16 ± 0.5	>0.05
9. Dizziness	0.31 ± 0.7	0.28 ± 0.8	0.31 ± 0.7	>0.05
10. Ear pain	0.08 ± 0.3	0.00 ± 0	0.08 ± 0.4	>0.05
11. Facial pain/pressure	0.40 ± 1	0.28 ± 1	0.42 ± 1	>0.05
12. Loss of smell or taste	0.42 ± 1.1	2.78 ± 0.9	0.14 ± 0.7	0.000*
13. Difficulty falling asleep	0.55 ± 1.1	0.94 ± 1.3	0.50 ± 1.1	>0.05
14. Waking up at night	0.63 ± 1.2	1.06 ± 1.3	0.58 ± 1.2	0.029*
15. Lack of a good night’s sleep	0.74 ± 1.3	1.17 ± 1.5	0.68 ± 1.3	>0.05
16. Waking up tired	0.88 ± 1.4	1.22 ± 1.4	0.84 ± 1.3	>0.05
17. Fatigue	0.91 ± 1.4	1.39 ± 1.5	0.85 ± 1.3	>0.05
18. Reduced productivity	0.49 ± 1	0.61 ± 1	0.47 ± 1	>0.05
19. Reduced concentration	0.48 ± 1	0.78 ± 1.3	0.44 ± 1	>0.05
20. Frustrated/restless/irritable	0.47 ± 1	0.78 ± 1.3	0.44 ± 0.9	>0.05
21. Sad	0.87 ± 1.3	1.11 ± 1.4	0.84 ± 1.3	>0.05
22. Embarrassed	0.95 ± 1.3	1.11 ± 1.4	0.93 ± 1.3	>0.05
TOTAL	11.61 ± 13.2	20.0 ± 13.4	10.62 ± 13	0.001*

### 3.3. Psychophysical olfactory evaluation

Objective olfactory evaluation with Sniffin’ sticks test battery was performed to 25 volunteered patients, 52% of whom (n: 13) were female and 48% of whom (n: 12) were male. The median age of the patients was 38 years (min–max: 23–52). Twenty-two patients were in group 1 and three patients were in group 2a. Twenty-four percent of the patients had a previous diagnosis of allergic rhinitis and 48% were smokers. None of the participants had self-reported olfactory dysfunction at the time of Sniffin’ sticks test. Only 2 patients reported olfactory dysfunction due to COVID-19 and both claimed to be totally recovered. 

The median SNOT-22 score of these 25 patients was 9 (min–max: 0–59). The median T, D, I, and TDI scores were 10.33 (min–max: 1–16), 10 (min–max: 6–16), 11 (min–max: 8–15), and 32 (min–max: 19.33–43), respectively. Sixty-four percent of the patients (n: 16) were normosmic and 36% (n: 9) were hyposmic. Eleven-point-one percent (n: 1) of the hyposmic patients had self-reported olfactory dysfunction due to COVID-19. Eighty-one-point-three percent of normosmic patients were in group 1. All the hyposmic patients were in group 1. Sixty-three-point-six percent of the hyposmic patients and 42.1% of tne normosmic patients were smokers. Twenty-five percent of normosmic patients and 22.2% of normosmic patients were diagnosed with allergic rhinitis. Smoking habit and previous diagnosis of allergic rhinitis did not differ significantly between hyposmic and normosmic patients. Demographic and clinical information of the patients participated in Sniffin’ Stick Test was summarized in Table 5.

**Table 5:** TDemographic and clinical information of the patients participated in Sniffin’ stick test.

	Normosmic(n:16)	Hyposmic(n:9)
Age median (min–max)	38 (25–52)	40 (23–50)
Sex		
Male	43.8%	55.6%
Female	56.3%	44.4%
Smoking	42.1%	63.6%
Allergic rhinitis	25%	22.2%
Disease group		
Group 1	81.3%	100%
Group 2	18.8%	–
Self-reported olfactory dysfunction	6.3%	11.1%
SNOT-22-median (min–max)	8.5 (0–59)	13 (0–22)

## 4. Discussion 

Anosmia is a well-known symptom of viral upper respiratory tract infections. A viral infection is the reason of anosmia in 40% of the cases in adult patients [9]. Many viruses such as rhinovirus, Epstein-Barr virus, and parainfluenza may cause mechanical obstruction with mucosal inflammation and rhinorrhea, resulting in olfactory dysfunction [10,11]. However, the smell disorder associated with COVID-19 has a different pathogenesis that can occur without rhinorrhea and nasal obstruction [4,8]. Lechien et al. claimed that 79.7% of COVID-19 patients with anosmia or hyposmia did not complain about rhinorrhea and nasal obstruction [4]. On the other hand, in our study, the mean score of “nasal obstruction” (question-2 of SNOT-22) of the patients with self-reported olfactory dysfunction was significantly higher than the patients without. 

In the literature, rates of olfactory dysfunction vary among studies between 3.2% and 98.3% [19,20] and gustatory dysfunction varies between 5.6% and 88% [3,4]. In a metaanalysis reported by Agyeman et al., olfactory dysfunction and gustatory dysfunction rates were found to be 41.0% (95% CI, 28.5% to 53.9%) and 38.2% (95% CI, 24.0% to 53.6%) respectively [21]. In our study we found that rates of olfactory and gustatory dysfunctions were both 10.5%. We attribute our low rates of olfactory and gustatory dysfunctions to the fact that most of the participants in the study were asymptomatic patients diagnosed during contact tracing.

COVID-19 may present in a wide clinical spectrum, from asymptomatic cases to severe illness, with or without pneumonia [22]. In our study, the patients were clustered in three groups according to disease severity, 73.1% of the patients were in group 1 (asymptomatic other than smell or taste dysfunction), 20.5% were in group 2a (symptomatic disease without pneumonia) and 6.4% were in group 2b (presented with pneumonia). The patients in group 1 had a lower rate of olfactory dysfunction compared to group 2 but that was not statistically significant. Several authors reported lower olfactory disorder rates in severe COVID-19 [23–26]. However, in the studies that used psychophysical olfactory tests, no relationship between disease severity and olfactory dysfunction was found [8,20,26,27].

Some studies reported significantly higher olfactory dysfunction rates in females than in males [4,28,29]. In our study, the rate of self-reported olfactory dysfunction was higher in females (13% vs. 7%) but that was not statistically significant. The relation between smoking and self-reported olfactory dysfunction varies among studies [24,30]. In our study, the rate of olfactory dysfunction of smokers was similar to that of nonsmokers. The mean ages of the patients with and without self-reported olfactory dysfunction were 39.5 (min–max: 18–55) and 36 (min–max: 19–71) respectively. Vaira et al. evaluated a large group of patients with psychophysical olfactory tests and did not find a relationship between age, sex, smoking, and olfactory dysfunction [31].

In our study, gustatory dysfunction rate of smokers was significantly higher than that of nonsmokers. No information was found in the literature on how smoking affects the susceptibility to gustatory dysfunction in COVID-19 patients. On the other hand, smoking is known to cause alternations of gustatory function [32]. 

All but one patient recovered from olfactory dysfunction in median time of 7 days (min–max: 0–30). One patient was still suffering from olfactory dysfunction 45 days after diagnosis. Gustatory dysfunction resolved in 16 of the patients within a median time of 8.5 days (min–max: 1–30). These findings were in line with the literature. In the study by Lechien et al. 96.7% of the patients recovered in two weeks [4]. Klepfenstein et al. pointed out that the average duration of anosmia was 8.9 days and ≥14 days for 20% of individuals [33]. Lee et al. reported that patients with olfactory or gustatory dysfunctions recovered within 3 weeks; with the average recovery time of 7 days [29].

SNOT-22 score of group 2a was higher than the others. Lechien et al. also reported higher SNOT-22 scores in the patients with moderate disease than patients with mild or severe disease [34]. Since the first 12 questions of SNOT-22 mostly cover the symptoms of upper respiratory tract infection, it was not surprising to have higher SNOT-22 scores in patients with moderate symptoms without pulmonary disease. Samaranayake et al. pointed out that “nasal blockage” (question 2) and “runny nose” (question 4) were more prevalent in patients with mild or moderate disease than patients with severe disease [35]. 

In the second step of our study, we aimed to evaluate olfactory function of recovered patients with sniffin’ sticks test battery. All the tests were performed at least 30 days after the diagnosis. No participant reported olfactory dysfunction at the time of test. According to TDI scores, 64% of the patients were normosmic and 36% were hyposmic. Moein et al. reported that nearly 65% of the patients were unaware of their olfactory dysfunction [20]. Vaira et al. reported that 14.5% of the patients without self-reported olfactory dysfunction were actually hyposmic [31]. On the other hand, being unaware of olfactory dysfunction is not rare with a prevalence of %22 in normal population [36]. According to this information, it may be wrong to say that hyposmia in our patients is caused by COVID-19, but it could be speculated that olfactory dysfunction is more common in COVID patients than patients’ self-report. 

There was not any significant difference of age, sex, severity of disease, and diagnosis of allergic rhinitis between normosmic and hyposmic patients. The median SNOT-22 score was higher in hyposmic patients. 

This study has several limitations. Firstly, both the survey and psychophysical olfactory tests were performed after the patients’ recovery. The survey was based on patients’ self-reported data. This posed a risk for recall bias. Secondly, almost all the patients who participated in psychophysical olfactory tests were those who did not report olfactory dysfunction during COVID-19. Therefore, no comment could be made on the permanence of the olfactory dysfunction caused by COVID-19. A third limitation was that the olfactory functions of the patients who underwent psychophysical olfactory tests were not objectively known prior to disease. Therefore, it could not be clarified whether the hyposmia in our patients was due to COVID or not.

## 5. Conclusion

Olfactory and gustatory dysfunctions have been a remarkable issue for physicians since the beginning of the COVID-19 outbreak. These symptoms occur in the early period of the disease. Olfactory and gustatory dysfunctions are more common in patients who are clinically symptomatic than those diagnosed during contact tracing. Olfactory dysfunction is not related to the severity of the disease. Objective tests may show that frequency of olfactory dysfunction is greater than frequency of self-reported olfactory dysfunction.

## Informed consent

The study was approved by the local ethical committee of Gazi University. All the participants provided informed consent.
